# Optimizing Indocyanine Green Dosage for Near-Infrared Fluorescence Perfusion Assessment in Bowel Anastomosis: A Prospective, Systematic Dose-Ranging Study

**DOI:** 10.3390/life14020186

**Published:** 2024-01-26

**Authors:** Leonard A. Lobbes, Katharina Schier, Kasper Tiebie, Nelly Scheidel, Ioannis Pozios, Richelle J. M. Hoveling, Benjamin Weixler

**Affiliations:** 1 Department of General and Visceral Surgery, Charité—Universitätsmedizin Berlin, Corporate Member of Freie Universität Berlin and Humboldt-Universität zu Berlin, Hindenburgdamm 30, 12203 Berlin, Germanyioannis.pozios@charite.de (I.P.);; 2 Quest Medical Imaging, Westrak 3, 1771 SR Wieringerwerf, The Netherlands

**Keywords:** indocyanine green, near-infrared fluorescence, dosage, perfusion, anastomosis, assessment, intraoperative visualization, bowel anastomosis, ileostomy reversal, ileostomy closure

## Abstract

Background: Indocyanine green (ICG) near-infrared fluorescence (NIRF) has emerged as a promising technique for visualizing tissue perfusion. However, within the wide range of dosages and imaging conditions currently being applied, the optimal dosage of ICG remains unclear. This study aimed to investigate the feasibility and implications of implementing lower dosages of ICG than commonly used for visual and quantitative perfusion assessment in a standardized setting. Methods: A prospective single-center cohort study was conducted on patients undergoing ileostomy reversal by hand-sewn anastomosis. ICG-NIRF visualization was performed before (T1) and after (T2) anastomosis with one of four different dosages of ICG (5 mg, 2.5 mg, 1.25 mg, or 0.625 mg) and recorded. Postoperatively, each visualization was evaluated for signal strength, completeness, and homogeneity of fluorescence. Additionally, perfusion graphs were generated by a software-based quantitative perfusion assessment, allowing an analysis of perfusion parameters. Statistical analysis comparing the effect of the investigated dosages on these parameters was performed. Results: In total, 40 patients were investigated. Visual evaluation demonstrated strong, complete, and homogeneous fluorescence signals across all dosages. Perfusion graph assessment revealed a consistent shape for all dosages (ingress followed by egress phase). While the average signal intensity decreased with dosage, it was sufficient to enable perfusion assessment even at the lowest dosages of 1.25 mg and 0.625 mg of ICG. The baseline intensity at T2 (the second intraoperative visualization) significantly decreased with dosage. The slope of the egress phase steepened with decreasing dosage. Conclusions: Lower dosages of ICG were sufficient for intraoperative perfusion assessment, while causing lower residual fluorescence and quicker egress in subsequent visualizations.

## 1. Introduction

The intraoperative assessment of tissue perfusion is of immense importance in surgical decision-making, particularly in procedures involving bowel anastomosis. Accurate assessment techniques allow informed decisions regarding the viability of tissues, potential complications, and the overall success of a procedure.

One of the most promising recently developed techniques is indocyanine green (ICG) near-infrared fluorescence (NIRF), which is currently increasingly being applied across all surgical fields [1,2,3,4]. ICG-NIRF has gained recognition as a reliable real-time technique for visualizing tissue perfusion, e.g., enabling the precise placement of anastomoses and the identification of potential vascular compromise, potentially reducing perfusion-related complications [5,6].

While the potential benefits of ICG-NIRF are well-established and its industrial implementation is rapidly progressing, there is considerable inhomogeneity in the devices used, the intraoperative imaging environment applied (e.g., distance of the NIRF camera to the imaging site, presence of surrounding light, timing of the injection, etc.), and, importantly, the dosage of ICG, which remains a topic of debate. Previous literature reports applied dosages of ICG per intraoperative visualization ranging from 2.5 milligrams (mg) to more than 15 mg as a bolus, or 0.1 mg to 0.3 mg per kilogram (kg) bodyweight [7,8,9,10,11,12,13]. To date, for anastomotic perfusion assessment, a dosage of 5 to 10 mg of ICG per visualization is recommended by an expert consensus and seems to be widespread [8].

The suitability of lower doses of ICG for NIRF visualization remains unknown, as does the effect of altering the dosage on both subjective visual and objective, software-based perfusion assessment. This is mainly due to the considerable differences in the devices, degrees of imaging environment standardization, and large range of different procedures included into existing studies [14,15]. Lower ICG dosages are likely to improve the accuracy when multiple subsequent intraoperative ICG-NIRF visualizations are performed (such as in anastomotic assessment), besides improving patient safety and reducing costs.

This study aimed to investigate the feasibility of dosage-reduction and the effect of dose-ranging on both the subjective visual and objective software-based assessment of intraoperative ICG-NIRF perfusion visualization in a comparable imaging environment for a single, homogenous surgical procedure to optimize ICG dosage for NIRF perfusion assessment in the future. The primary goal was to assess the potential for dose reduction without compromising the ability to evaluate perfusion patterns during surgical procedures.

## 2. Materials and Methods

### 2.1. Study Design

We performed a prospective single-center cohort study (NCT04709445) from May 2020 to February 2022 at the Department of General and Visceral Surgery, Charité University Medicine Berlin, Campus Benjamin Franklin (tertiary referral center). Study approval was obtained by the Charité University Hospital Ethics Committee (No. EA1/087/20), and all data were reported in conformity with the STROBE guidelines [16].

Patients undergoing ileostomy reversal by hand-sewn end-to-end and side-to-end anastomosis were included.

### 2.2. Patient Enrollment

A total of 40 patients undergoing ileostomy reversal were enrolled in the study. Study inclusion required an indication for ileostomy reversal, an age ≥ 18 years, and an American Society of Anesthesiologists (ASA) physical status ≤ 3. Patients were excluded in case of iodine allergy, hypersensitivity to ICG or sodium iodide, hyperthyroidism, thyroid nodules, a previously poorly tolerated injection of ICG, pregnancy or breastfeeding, a coexisting malignancy, or liver dysfunction (with a Model End Score Liver Disease (MELD) score > 10). Informed consent was obtained from all participants prior to their inclusion at least 24 h before surgery.

### 2.3. Surgical Procedure

Following the operative standard of our tertiary referral center, ileostomy reversal was performed by open surgery. After surrounding circular incision, adhesiolysis was performed, followed by marginal resection on the oral and aboral ileostomy site. Anastomotic technique was standardly hand-sewn as an end-to-end anastomosis.

### 2.4. Intraoperative ICG-NIRF Imaging

**Figure 1:**
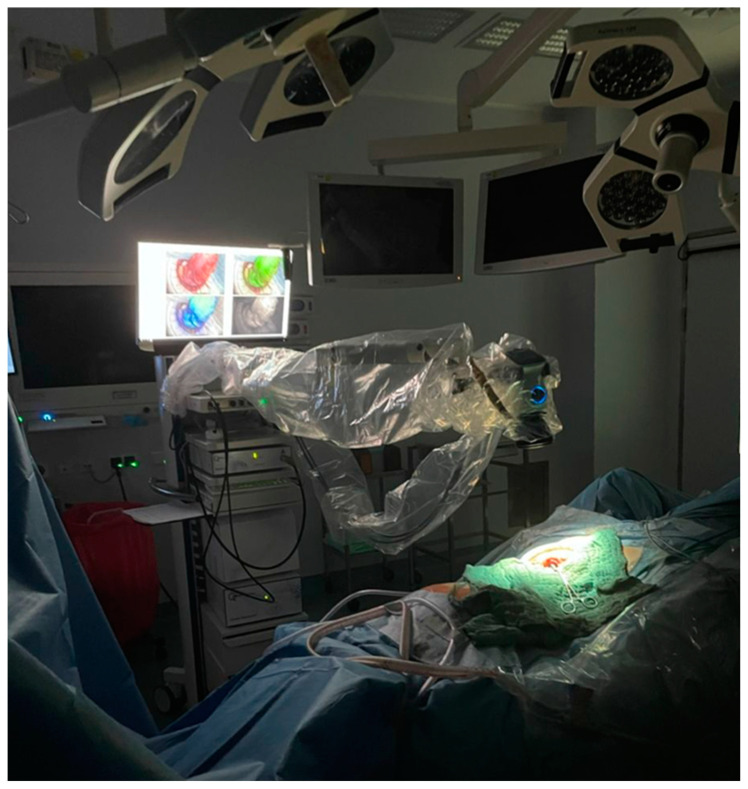
Intraoperative imaging environment. A standardized imaging environment was established for intraoperative procedures. The Quest Spectrum Camera System was positioned on the left side of the patient and secured using a movable arm, which allowed for adjustments in camera position, angle, and focus. The camera was covered with sterile drapes to maintain aseptic conditions. To ensure accurate imaging, all external light sources, including surrounding and surgical lights in the darkened operating room (OR), were turned off during the 120-s measurement period. This ensured that the camera was the only source of emitted light. The distance between the camera and the imaging site was documented. Surgical drapes were placed around the imaging site as a negative control. Detailed records of intraoperative clinical data, camera settings, and the imaging environment were meticulously maintained during all visualizations. This setup was designed to capture high-quality recordings while minimizing potential confounding factors.

The Quest Spectrum^®^ imaging system was used for intraoperative imaging at two time points: before (time point T1) and after (time point T2) anastomosis formation. The camera was positioned on the left side of the patient and securely fixed with a movable arm. Sterile drapes were employed to maintain aseptic conditions, and surgical drapes were placed around the imaging site as a negative control. Sources of surrounding light were consistently removed and blocked from the operating room. Camera settings were standardized at the same exposure time (50 ms) and the AutoColor mode deactivated. The distance between camera and imaging site were recorded to ensure a standardized imaging environment (Figure 1). ICG (VerDye, Diagnostic Green GmbH, Aschheim, Germany, 25 mg) was dissolved in the respective amount of sterile water to yield a 0.625 mg/mL to 5 mg/mL concentration, respectively, and administered intravenously as a bolus of the respective dosage at both time points. Each ICG-NIRF visualization was video recorded for postoperative evaluation, starting at the moment of ICG injection to allow for observation of signal acquisition and of residual fluorescence in case of a previous visualization.

### 2.5. ICG Dosage Selection

Four different dosages of ICG were evaluated per visualization for 10 patients each: 5 mg, 2.5 mg, 1.25 mg, and 0.625 mg. The selection of these dosages was based on previous literature and clinical experience, aiming to assess the feasibility of using lower dosages for bowel perfusion assessment.

### 2.6. Comprehensive, Blinded Subjective Evaluation

Postoperatively, each ICG-NIRF video recording was evaluated independently by four observers with a wide range of clinical experience: researcher, surgical resident, board-certified surgeon, and attending surgeon. The observers independently assessed the recordings without knowledge of the dosage administered. The NIRF-visualization and video recording included four different visualization modes: visible light mode, fluorescence mode, green overlay mode (of fluorescence and visible light) and heat map overlay mode (of fluorescence and visible light). For accuracy, the observers assessed solely the fluorescence mode of the recording for the following: signal strength (0 = no signal; 1 = detectable signal; 2 = strong signal), completeness of fluorescence at anastomotic site (yes = 1; no = 2), and the homogeneity of fluorescence (yes = 1; no = 2). For time point T2, additionally, the presence and strength of a residual signal from the previous visualization was evaluated (0 = no signal; 1 = detectable signal; 2 = strong signal).

### 2.7. Postoperative Software-Based Objective Perfusion Graph Assessment

**Figure 2:**
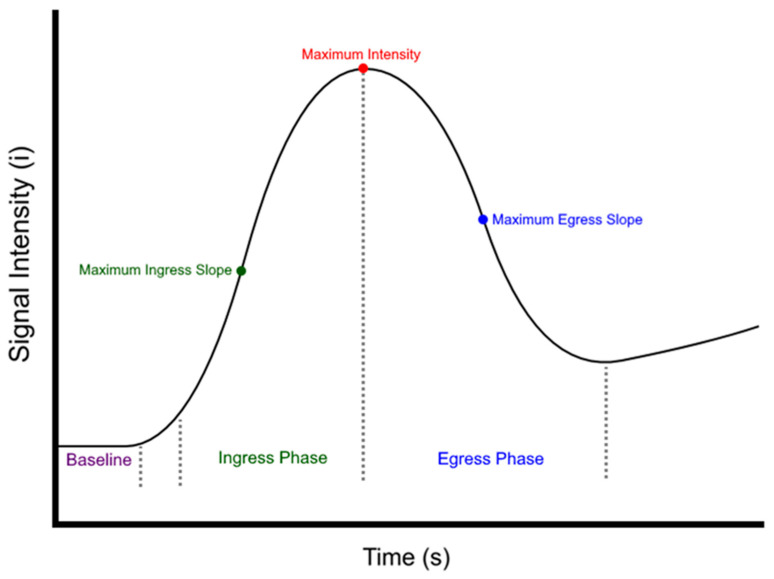
Perfusion graph assessment. A visual representation of the subsequent phases analyzed within the signal intensity curve. The baseline intensity was calculated using the average signal intensity between the start of the recording and the point where the intensity reached ≥ 1% of its maximum intensity value. The ingress phase started when the signal intensity reached ≥ 10% of its maximum intensity value and ended when the maximum intensity value was reached. The egress phase started at the maximum intensity value and ended when the slope of the curve was no longer negative. The signal phase consisted of both the ingress and egress phases.

Additionally, a software-based objective perfusion assessment of the video recordings was conducted using the Quest Research Software^TM^ (Version Number 4.6). The analysis involved examining the distribution of fluorescence intensity over time to generate a perfusion graph. To accomplish this, 9 regions of interest (ROIs) were strategically positioned in a standardized and predefined manner. These included 1 ROI as a negative control in a non-patient tissue area, 4 ROIs on the oral side of the anastomosis, and 4 ROIs on the aboral side of the anastomosis. Specifically, at least 2 ROIs on each side were placed directly at the (expected) anastomotic site, spaced evenly along the entire length. The remaining ROIs were positioned proximal/distal to the anastomotic site, aligned sequentially along the course of the bowel. Any instances where a ROI was situated in an area with fatty tissue or hematoma were noted to account for potential variations in ingress and egress curves. Perfusion curves were generated for each ROI by plotting fluorescence intensity over time, resulting in individual perfusion graphs for each recording. Utilizing the Quest Research Software^TM^, the quantitative perfusion assessment for each investigated dosage consisted of analyzing the following parameters of the perfusion graph: baseline intensity (before the beginning of the signal phase), average intensity during the signal phase (consisting of both inflow and outflow phase), average and maximum slope of the ingress (i.e., inflow phase) phase, as well as average and maximum slope of the egress (i.e., outflow phase) of the perfusion graph (Figure 2).

### 2.8. Statistical Analysis

Using the Kruskal–Wallis Test, the investigated characteristics of the perfusion graphs of the four dosage groups were compared to each other with the null hypothesis being that the medians of all groups are equal. In order to determine which of these groups differ from each other, the Dunn test was used as the posthoc test. As multiple testing was done, the *p*-values that resulted from this test were adjusted using the Benjamini & Hochberg false-discovery rate (FDR) correction. The chosen alpha level for the *p*-value to be statistically significant was set at 0.05 for both tests.

### 2.9. Sample Size

Due to the innovative nature of the assessment method used in this study, there was a lack of existing data to guide the determination of an optimal sample size. However, the findings of this study can serve as a foundation for future investigations, allowing for the possibility of conducting power analysis to determine an appropriate sample size for subsequent studies.

### 2.10. Clinical Data and Follow-Up

For accuracy and clinical context, detailed clinical follow-up was performed, including postoperative 30-day morbidity and anastomotic leak.

## 3. Results

### 3.1. Baseline Patient Characteristics

The baseline patient characteristics of the study population (*n* = 40) are summarized in Table 1. Among the patients, 19 were female and 21 were male. The distribution of ASA physical status was as follows: 4 patients classified as ASA I, 30 patients as ASA II, and 6 patients as ASA III. The mean age at the time of the operation was 42.8 years, with a median of 36.3 years and a range of 18.2 to 89.8 years. The average BMI was 22.95 kg/m^2^, with a median of 21.9 kg/m^2^ and a range of 14.9 to 32.7 kg/m^2^.

The most common underlying condition was ulcerative colitis (18 patients), followed by Crohn’s disease (12 patients), indeterminate colitis (1 patient), carcinoma of the rectum (3 patients), carcinoma of the colon (2 patients), perianal fistula (1 patient), colon ischemia (2 patients), and colon perforation (1 patient).

Each ICG dosage (0.625 mg, 1.25 mg, 2.5 mg, and 5 mg) was administered to 10 patients, respectively. All surgeries were performed using an open approach. The majority of anastomoses were performed end-to-end (39 patients), while one patient had a side-to-side anastomosis after ileal segmental resection of 6 cm due to individual anatomy. Two patients experienced an anastomotic leak within 30 days, while the remaining 38 patients did not.

These baseline patient characteristics provide an overview of the study population and set the context for interpreting the results.

### 3.2. Comprehensive, Subjective Evaluation of ICG-NIRF Visualization

**Figure 3:**
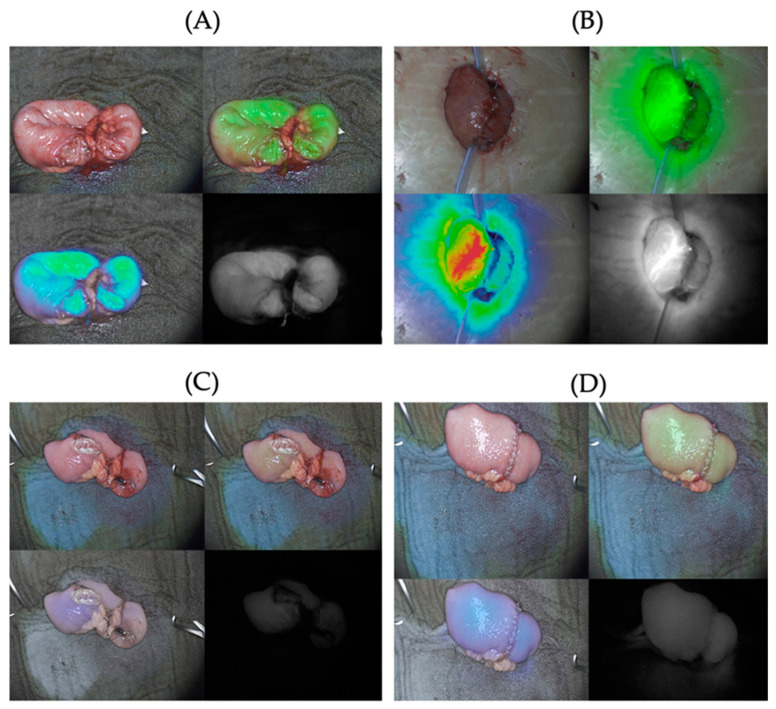
Fluorescence signal strengths in subjective evaluation before (time point T1) and after (time point T2) anastomosis. As shown intraoperatively by the Quest Spectrum™ system, each image consists of four visualization modes: visible-light mode (top left), green overlay mode (top right), heat map overlay mode (bottom left), and fluorescence mode (bottom right). For accuracy, in this study, only the fluorescence mode was postoperatively subjectively evaluated. The assessment at both time points includes the evaluation of signal strength (0–1–2), homogeneity, and completeness of fluorescence (yes–no). At time point T2, an additional evaluation is conducted to determine the persistence of signal strength from the previous measurement (0–1–2). (**A**) Example of signal strength 2 = strong at time point T1. (**B**) Example of signal strength 2 = strong at time point T2. (**C**) Example of signal strength 1 = detectable at time point T1. (**D**) Example of signal strength 1 = detectable at time point T2.

ICG-NIRF visualization and subjective evaluation of the ICG-NIRF signal were possible at all four dosages of ICG (Figure 3). Signal strength varied between 1 = detectable and 2 = strong signal at both time points. In case 1 and case 11, ICG-NIRF imaging was only performed at one time point, respectively, due to intraoperative surgical circumstances.

The results of the subjective individual evaluations of signal strength, completeness of fluorescence, and homogeneity of ICG-NIRF visualizations by the four independent observers at time point T1 (before anastomosis) were categorized by dosage (Table 2). The evaluation of signal strength (0 = no signal; 1 = detectable signal; 2 = strong signal) showed consistent assessments among the observers for most cases and dosages. The majority of cases were evaluated as having a strong signal (2 = strong signal) by all observers. There was 1 case of inter-observer variability for signal strength, (detectable = 1 vs. strong = 2) out of the 40 cases. For completeness of fluorescence at the anastomotic site (yes = 1, no = 2), most cases were evaluated as completely fluorescent (yes = 1). There were 2 cases with inter-observer variability (yes = 1 vs. no = 2). Similarly, the assessment of fluorescence homogeneity revealed a general consensus among the observers, as the majority of cases were determined to have homogeneous fluorescence (homogenous = 1). 6 cases showed inter-observer variability (homogenous = 1 vs. heterogenous = 2).

The results of the subjective individual evaluations at time point T2 were also categorized by dosage, additionally initially assessed for a residual signal after the first visualization (Table 3). Most cases showed a detectable or strong residual signal, as indicated by the values of 1 or 2, respectively. For lower dosages (1.25 mg and 0.625 mg), the residual signal was considerably weaker at just detectable (=1) or not present (=0). There was inter-observer variability in four cases.

The signal strength was generally strong (=2) across all cases including lower dosages, except for two instances where it was lower (=1) across all dosages. There was no inter-observer variability.

Completeness of fluorescence at the anastomotic site was found to be completely fluorescent (=1) in the majority of cases. Six cases were marked as not completely fluorescent (=2). There was inter-observer variability in three cases.

Fluorescence homogeneity was predominantly homogeneous (=1) in the majority of cases. One case was found to have a heterogenous signal (=2) as reported by observers. There was inter-observer variability in two cases.

Out of the 40 cases with 80 visualizations and 320 subjective evaluations in total, there was a relatively small overall inter-observer variability (11 instances for time point T1, 9 instances for time point T2) between the four independent observers with varying degrees of surgical experience. Overall, the results of the subjective evaluation of the ICG-NIRF visualizations indicated that at both time points, there was a strong, complete and homogenous fluorescence signal for all four investigated dosages of ICG, including low dosages of 1.25 mg and 0.625 mg. Notably, for these low ICG dosages of 1.25 mg and 0.625 mg, considerably lower or no residual fluorescence from the first visualization was found to be present at time point T2.

### 3.3. Quantitative Software-Based Assessment of Perfusion Graphs for Each Dosage

**Figure 4:**
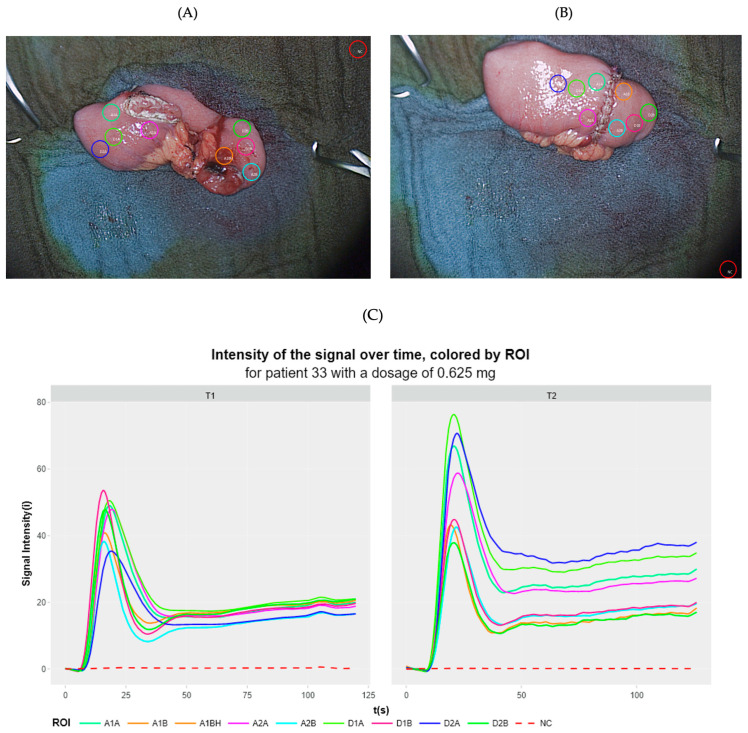
Objective assessment of fluorescence intensity over time for specific regions of interest (ROIs). For objective evaluation, specific regions of interest were predefined using the Quest Research Software^TM^. In the video recording of each ICG-NIRF visualization, a total of 9 strategically positioned regions of interest (ROIs) were delineated in a standardized and predefined manner. These ROIs consisted of 1 negative control ROI located in a non-patient tissue area, 4 ROIs on the oral side of the anastomosis, and 4 ROIs on the aboral side of the anastomosis. Specifically, to ensure comprehensive assessment, a minimum of 2 ROIs on each side were placed directly at the (expected) anastomotic site, evenly distributed along the entire length. The remaining ROIs were systematically positioned proximal/distal to the anastomotic site, sequentially following the course of the bowel. Any instances where an ROI intersected with fatty tissue or hematoma were carefully documented to account for potential deviations in perfusion curves. For each ROI, the fluorescence intensity (in i) was extracted and plotted on the *y*-axis against time (in seconds) on the *x*-axis, resulting in a perfusion curve per ROI comprising a perfusion graph per visualization. (**A**) ROI placement at time point T1. (**B**) ROI placement at time point T2. (**C**) Perfusion curves extracted from the ROIs at time point T1 (left) and time point T2 (right).

Utilizing the Quest Research Software™, the signal fluorescence intensity curves were extracted for each region of interest (ROI) (Figure 4) in both absolute intensity and as a percentage of the maximum fluorescence intensity value of the respective ROI. The former will be referred to as the non-normalized version (in i), and the latter will be referred to as the normalized version (in %). All curves were graphed against time (in seconds) to generate a perfusion curve for each ROI.

Preprocessing steps were performed on all curves by subtracting the baseline intensity and smoothing the curves. For the normalized version, the normalization step was performed between the baseline subtraction and the smoothing. The baseline intensity was subtracted from each of these curves using the average intensity from the start of the recording to where the signal intensity reached ≥1% of its maximum value. The smoothing was performed using the Whittaker–Henderson smoothing algorithm using a lambda value of 500,000.

**Figure 5:**
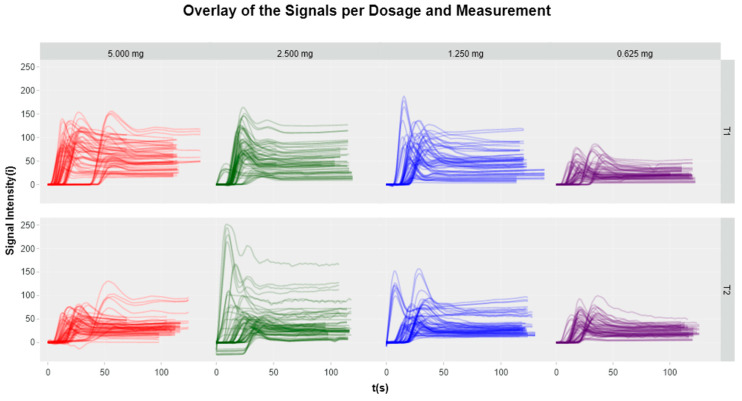
Plots of all perfusion graphs per dosage. Irrespective of the dosage, a consistent pattern was evident in the signal phase analysis. The perfusion curves exhibited an initial positive slope, indicating the ingress phase characterized by the inflow of blood. This was followed by a peak representing the maximum signal intensity, and finally, a subsequent negative slope indicating the egress phase corresponding to the outflow of blood.

It was possible to generate perfusion curves at all dosages, allowing an objective, quantitative perfusion assessment. Regardless of the dosage, a consistent pattern was observed: the signal phase consisted of an initial positive slope indicating the inflow of blood (ingress phase), followed by a peak representing the maximum signal intensity, and then a subsequent negative slope indicating the outflow of blood (egress phase) (Figure 5).

**Figure 6:**
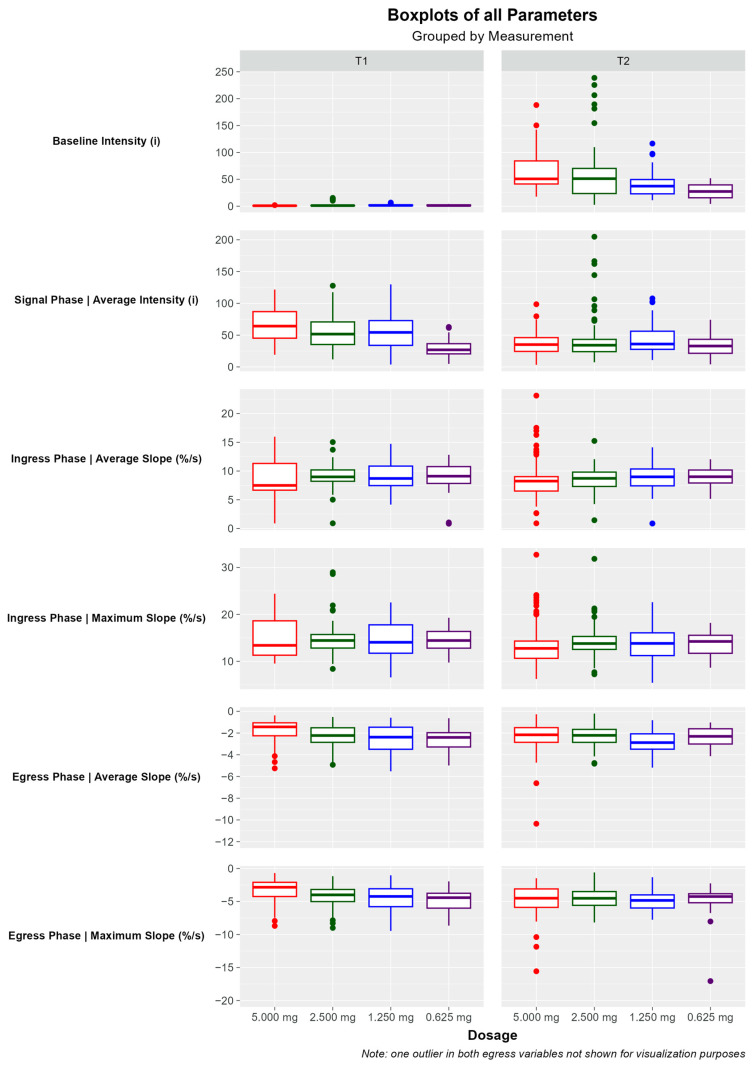
Boxplots showing the value for each parameter and each dosage at time point T1 and T2. For visibility, the scaling of average and maximum slope of the egress phase was adjusted. As a result, two outliers in total are not visible. They were not removed from the statistical analysis and calculation of the box plots.

For each dosage investigated, the baseline intensity, average intensity during the signal phase, average slope and maximum slope of the ingress phase, and the average slope and maximum slope of the egress were analyzed for both visualization time points. Boxplots were generated for all parameters (Figure 6).

#### 3.3.1. Baseline Intensity

The baseline intensity was near-zero at time point T1 for all dosages and decreased with decreasing dosage at time point T2 (*p*-value at T1 < 0.001, *p*-value at T2 < 0.001).

#### 3.3.2. Signal Phase Intensity

The average intensity of the signal phase showed a trend of decreasing with decreasing dosage at time point T1 (*p*-value < 0.001). At time point T2, this same trend was not observed (*p*-value = 0.114).

#### 3.3.3. Ingress Phase

The average slope of the ingress phase was approximately equal for all dosages (*p*-value at T1 = 0.068, *p*-value at T2 = 0.056). The maximum slope in the ingress phase did not vary significantly with dosage (*p*-value at T1 = 0.640, *p*-value at T2 = 0.172).

#### 3.3.4. Egress Phase

The average slope of the egress phase steepened with decreasing dosage at both time points (*p*-value at T1 < 0.001, *p*-value at T2 = 0.002 by Kruskal–Wallis test). The Dunn post-hoc test confirmed the difference when comparing the 5 mg dosage group with the other dosages for time point T1 (respective adjusted *p*-value for comparison of 5 mg to 0.625 mg < 0.001, for comparison of 5 mg to 1.25 mg = 0.001, for comparison of 5 mg to 2.5 mg = 0.018).

For the T2 time point, the average slope of the egress phase of the 1.25 mg dosage group was significantly steeper in comparison to the other dosages (respective adjusted *p*-value by Dunn post-hoc test for comparison of 1.25 mg to 0.625 mg = 0.016, for comparison of 1.25 mg to 2.5 mg = 0.003, for comparison of 1.25 mg to 5 mg = 0.005).

At time point T1, the maximum slope of the egress phase steepened with decreasing dosage (*p*-value at T1 < 0.001 by Kruskal–Wallis test). The Dunn post-hoc test confirmed the significant difference when comparing the 5 mg dosage group with the other dosages for time point T1 (respective adjusted *p*-value for comparison of 5 mg to 0.625 mg < 0.001, for comparison of 5 mg to 1.25 mg < 0.001, for comparison of 2.5 mg to 5 mg = 0.002). At time point T2, no significant difference in the maximum slope was detected with regards to dosage (*p*-value = 0.392 by Kruskal–Wallis test). Likewise, the Dunn post-hoc test did not indicate any significant differences with regards to dosage at time point T2.

### 3.4. Incidental Finding: Inhomogeneity of Fluorescence along Hematoma and Suture Lines

**Figure 7:**
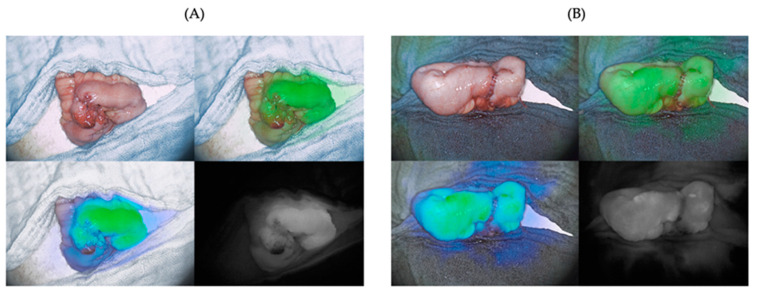
Subjective inhomogeneity of fluorescence along areas of hematoma and suture lines. Each image consists of four visualization modes: visible-light mode (top left), green overlay mode (top right), heat map overlay mode (bottom left), and fluorescence mode (bottom right). (**A**) shows an example where a hematoma led to an inhomogeneity, whereas in (**B**) the suture line is not as fluorescent as the rest of the bowel.

An additional, incidental finding was an inhomogeneity of fluorescence along areas of hematoma and suture lines (Figure 7). Hematoma was identified in four cases. Furthermore, in seven cases, the suture line exhibited reduced fluorescence compared to the surrounding bowel in the subjective evaluation.

## 4. Discussion

This study aimed to investigate the feasibility of dosage reduction and the effect of dose-ranging on subjective visual and objective software-based assessment of ICG-NIRF perfusion visualization in a standardized imaging environment.

The subjective evaluation of ICG-NIRF visualizations by independent observers indicated that all investigated dosages, including lower dosages of 1.25 mg and 0.625 mg, provided strong, complete, and homogeneous fluorescence signals. This suggests that even at lower dosages, ICG-NIRF visualization is feasible and reliable for assessing perfusion patterns during surgical procedures. Notably, lower dosages demonstrated considerably lower or no residual fluorescence from previous visualizations, which may enhance accuracy in subsequent intraoperative assessments.

The software-based quantitative analysis of perfusion graphs generated from the ICG-NIRF visualizations revealed a consistent pattern regardless of the dosage administered. The signal phase exhibited an initial positive slope (ingress phase), followed by a peak representing maximum signal intensity, and a subsequent negative slope (egress phase).

Statistical analysis revealed that the baseline intensity at time point T2 (after the initial visualization) was significantly lower with lower dosages. This implies that lower dosages result in a reduced background fluorescence, enhancing the clarity of perfusion visualization for multiple intraoperative visualizations, as typically used for anastomotic assessment.

While the average signal intensity decreased with dosage, it was sufficient to enable quantitative perfusion assessment at even the lowest dosages of 0.625 mg and 1.25 mg of ICG without affecting the shape of the perfusion curves. This was substantiated by the fact that no significant differences were found in the ingress phase with regards to dosage for both visualization time points. For the egress phase, a significantly steeper average and maximum slope were observed in the 5 mg dosage group at time point T1 and a steeper average slope for the 1.25 mg dosage at time point T2. The other dosage groups did not exhibit a significant difference.

One reason for the decreased average and maximum slope of the egress phase at a dosage of 5 mg in comparison to the other dosages could be the occurrence of accumulation and diffusion. At the comparably higher dosage, the amount of ICG leaving the imaging site during egress seems to be reduced. Additionally, self-quenching, a property of ICG at higher concentrations resulting in reduced fluorescence intensity due to overlap between absorption and emission [17,18] could play a role. The steeper average slope of the egress at 1.25 mg suggests an increased or quickened egress, which would be beneficial for multiple assessments. These are interesting findings that require further investigation with a greater sample size to reassess statistical significance. This could reveal quenching at dosages currently commonly used for in-vivo fluorescence imaging, possibly due to imaging environment interactions at the molecular level.

The reduction in signal intensity and baseline fluorescence with lower dosages can be advantageous in multiple ways. First, it may enhance accuracy when multiple subsequent ICG-NIRF visualizations are performed, as the residual fluorescence from previous visualizations is minimized. This allows for clearer differentiation between perfusion patterns during successive assessments. Second, lower dosages may improve patient safety by minimizing the risk of adverse effects associated with higher ICG dosages. Additionally, the use of lower dosages can potentially reduce costs associated with ICG administration.

Interestingly, the statistical analysis showed a significant difference in signal intensity and baseline fluorescence among different dosages. However, it is important to consider the clinical significance of these differences. Despite the statistical significance, the signal intensity at lower dosages was still sufficient for perfusion assessment, suggesting that these differences may not have substantial clinical implications. The focus of ICG-NIRF imaging should be on the optimal dosage and imaging environment for software-based quantitative, objective perfusion assessment methods rather than the visual signal intensity, as previously reported [19,20,21,22].

An additional, incidental finding of our study was the subjectively observed reduced fluorescence in areas of hematoma (4 cases) and along suture lines after completed anastomosis (7 cases). These findings suggest that suture material or the presence of blood may contribute to subjectively interpreted fluorescence inhomogeneity, possibly also perfusion impairment. Importantly, these subjectively perceived inhomogeneities did not show any differences in the quantitative, software-based perfusion assessment, indicating greater accuracy of the latter. Further research is needed to investigate the implications of these inhomogeneities on the clinical interpretation of fluorescence imaging to optimize and implement quantitative techniques.

This study has some limitations that should be acknowledged. Firstly, it focused on small bowel anastomoses (ileostomy reversal) and may not fully represent the findings in other surgical contexts. However, the homogeneity and high degree of standardization in the investigated procedure provided optimal conditions for a dose-ranging study, minimizing the effect of intraoperative confounders. Secondly, the sample size was relatively small, and the lack of existing data made it challenging to determine an optimal sample size. There were some outliers, evident in Figure 6, mainly due to imaging conditions and movement, which were nonetheless included in the quantitative analysis for validity. In future studies, we aim to further investigate and implement the standardized imaging environment, consider a larger sample size, and include different surgical procedures to validate and generalize the findings.

Additionally, this study focused solely on the usage of ICG in perfusion assessment, whereas the application of ICG is widespread in multiple other settings, for example, in sentinel lymph node detection, tumor visualization, or lymphatic mapping. The dosages recommended in this study can thus solely be applied to perfusion evaluation. Currently, there are no standardized application methods for ICG usage in the different clinical contexts, although most studies are using dosages in a range similar to the range used for perfusion assessment [1,2]. Moreover, a widespread heterogeneity can be observed in the literature regarding the timepoint and localization of ICG application [1,23]. Separate studies need to be conducted for each setting, focusing on potential differences in dosage and application of ICG in different surgical contexts, as already partly conducted by our group [20,21,22,24]. Additionally, our methodology could be combined with pulse spectrophotometry of ICG and blood samples to evaluate ICG clearance and predict a curve of metabolism and clearance versus time for ICG imaging.

Compared to the existing literature, we have investigated considerably lower dosages in a comparable intraoperative setting. De Nardi et al. conducted a multicenter randomized controlled trial evaluating ICG angiography for assessing anastomosis perfusion in laparoscopic colorectal resections, using considerably higher dosages of 0.3 mg/kg body weight [25]. Iguchi et al. investigated the utility of ICG fluorescence imaging for assessing intestinal perfusion during intracorporeal colorectal anastomosis, using a dosage of 12.5 mg ICG per visualization [26].

The notion of dosage reduction is supported by previous literature. Van Manen et al. provided a comprehensive guide for the use of ICG and methylene blue in fluorescence-guided abdominal surgery, recommending a dosage of 2.5 mg ICG per visualization [13]. Dip et al. published consensus guidelines on the general use of near-infrared fluorescence imaging and ICG-guided surgery, suggesting that lower dosages have been used intraoperatively but have not been investigated systematically [11].

Overall, the literature expresses a demand for dosage and imaging environment optimization and standardization for the application of ICG-NIRF in the future, as seen in the recently published consensus on ICG-guided surgery by the European Association for Endoscopic Surgery [27]. The findings of our study contribute by specifically investigating a range of ICG dosages on both comparable NIRF visualizations and quantitative perfusion assessments. Our findings demonstrate that lower dosages of ICG, as low as 0.625 mg per visualization, are sufficient for generating perfusion graphs and evaluating perfusion patterns during surgical procedures without compromising the ability to assess tissue perfusion.

The dosages used in this study (0.625 mg, 1.25 mg, 2.5 mg, and 5 mg) were selected based on previous literature, aiming to investigate considerably lower dosages than the commonly recommended dosages for anastomotic perfusion assessment (5 to 10 mg and more) [8]. Our study demonstrates that lower dosages can provide comparable results in terms of perfusion assessment parameters, suggesting the potential for dose reduction without compromising the accuracy of the technique.

## 5. Conclusions

The findings of this study revealed that lower dosages of ICG than currently widely used are sufficient for perfusion assessment, offering potential advantages in terms of accuracy, patient safety, and cost-effectiveness. These findings support the reduction and optimization of ICG dosage for NIRF perfusion assessment and emphasize the importance of standardized imaging environments, dosing protocols and quantitative methods of fluorescence signal assessment to ensure consistent and reliable results in the future. Further research, including larger studies and different surgical contexts, is urgently necessary to validate these findings and establish widely accepted guidelines for the optimal use of ICG-NIRF in surgical practice.

## Figures and Tables

**Table 1:** Baseline patient data.

Characteristic		All Patients (*n* = 40)
Sex	female	19
	male	21
ASA physical status	I	4
	II	30
	III	6
Age at operation (years)	mean	42.8
	median	36.3
	range	18.2–89.8
BMI (kg/m^2^)	mean	22.95
	median	21.9
	range	14.9–32.7
Underlying condition	ulcerative colitis	18
	Crohn’s disease	12
	indeterminate colitis	1
	carcinoma of the rectum	3
	carcinoma of the colon	2
	perianal fistula	1
	colon ischemia	2
	colon perforation	1
ICG dosage (mg)	5	10
	2.5	10
	1.25	10
	0.625	10
Type of surgery	open	40
	laparoscopic	0
Type of anastomosis	end-to-end	39
	side-to-side	1
Anastomotic leak	yes	2
	no	38

Abbreviations: *n* = number; ASA = American Society of Anesthesiologists; BMI = body mass index; kg = kilograms; m^2^ = square meters; ICG = indocyanine green dye.

**Table 2:** Inter-observer consistency of subjective ICG-NIRF evaluation per dosage at time point T1 (before anastomosis).

		Signal Strength (0 = No Signal; 1 = Detectable Signal; 2 = Strong Signal)				Completeness of Fluorescence at Anastomotic Site (yes = 1, no = 2)				Fluorescence Homogeneity (Homogenous = 1, Heterogenous = 2)			
Case No.	ICG Dosage (mg)	Observer No.											
		1	2	3	4	1	2	3	4	1	2	3	4
1	5	2	2	2	2	1	1	1	1	1	1	1	1
2		2	2	2	2	1	1	1	1	1	1	1	1
3		2	2	2	2	2	2	2	2	**2**	**1**	**1**	**1**
4		2	2	2	2	1	1	1	1	**1**	**1**	**2**	**1**
5		2	2	2	2	1	1	1	1	1	1	1	1
6		2	2	2	2	1	1	1	1	1	1	1	1
7		2	2	2	2	1	1	1	1	1	1	1	1
8		2	2	2	2	1	1	1	1	1	1	1	1
9		2	2	2	2	1	1	1	1	1	1	1	1
10		2	2	2	2	1	1	1	1	1	1	1	1
11	2.5	-				-				-			
12		2	2	2	2	1	1	1	1	**2**	**1**	**1**	**1**
13		**2**	**2**	**1**	**1**	1	1	1	1	**2**	**1**	**1**	**1**
14		2	2	2	2	1	1	1	1	1	1	1	1
15		2	2	2	2	1	1	1	1	1	1	1	1
16		2	2	2	2	1	1	1	1	1	1	1	1
17		2	2	2	2	1	1	1	1	1	1	1	1
18		2	2	2	2	1	1	1	1	1	1	1	1
19		2	2	2	2	1	1	1	1	1	1	1	1
20		1	1	1	1	**1**	**1**	**2**	**1**	1	1	2	1
21	1.25	2	2	2	2	1	1	1	1	1	1	1	1
22		1	1	1	1	2	2	2	2	**2**	**1**	**2**	**1**
23		2	2	2	2	1	1	1	1	1	1	1	1
24		2	2	2	2	1	1	1	1	1	1	1	1
25		2	2	2	2	1	1	1	1	1	1	1	1
26		2	2	2	2	1	1	1	1	1	1	1	1
27		2	2	2	2	1	1	1	1	1	1	1	1
28		2	2	2	2	1	1	1	1	1	1	1	1
29		2	2	2	2	1	1	1	1	1	1	1	1
30		2	2	2	2	1	1	1	1	1	1	1	1
31	0.625	1	1	1	1	**2**	**1**	**2**	**1**	**2**	**1**	**2**	**1**
32		2	2	2	2	1	1	1	1	1	1	1	1
33		**1**	**1**	**2**	**2**	1	1	1	1	1	1	1	1
34		2	2	2	2	1	1	1	1	1	1	1	1
35		2	2	2	2	1	1	1	1	1	1	1	1
36		2	2	2	2	1	1	1	1	1	1	1	1
37		1	1	1	1	1	1	1	1	1	1	1	1
38		2	2	2	2	1	1	1	1	1	1	1	1
39		2	2	2	2	1	1	1	1	1	1	1	1
40		2	2	2	2	1	1	1	1	1	1	1	1

Observer No.: 1 = researcher, 2 = surgical resident, 3 = board-certified surgeon, 4 = attending surgeon; (-) = no visualization available due to intraoperative circumstances. Cases of inter-observer variability in fluorescence signal evaluation are marked as bold on green background.

**Table 3:** Inter-observer consistency of subjective ICG-NIRF evaluation per dosage at time point T2 (after anastomosis).

		Residual Signal (0 = No Signal; 1 = Detectable Signal; 2 = Strong Signal)				Signal Strength (0–1–2)				Completeness of Fluorescence at Anastomotic Site (yes = 1, no = 2)				Fluorescence Homogeneity (Homogenous = 1, Heterogenous = 2)			
Case No.	ICG Dosage (mg)	Observer No.															
		1	2	3	4	1	2	3	4	1	2	3	4	1	2	3	4
1	5	-				-				-				-			
2		2	2	2	2	2	2	2	2	1	1	1	1	**2**	**1**	**1**	**1**
3		1	1	1	1	2	2	2	2	1	1	1	1	1	1	1	1
4		**2**	**1**	**1**	**2**	2	2	2	2	1	1	1	1	1	1	1	1
5		1	1	1	1	2	2	2	2	2	2	2	2	1	1	1	1
6		1	1	1	1	2	2	2	2	1	1	1	1	1	1	1	1
7		1	1	1	1	2	2	2	2	2	2	2	2	1	1	1	1
8		1	1	1	1	2	2	2	2	1	1	1	1	1	1	1	1
9		2	2	2	2	2	2	2	2	1	1	1	1	1	1	1	1
10		1	1	1	1	2	2	2	2	**1**	**2**	**1**	**1**	1	1	1	1
11	2.5	-				2	2	2	2	1	1	1	1	2	2	2	2
12		1	1	1	1	2	2	2	2	1	1	1	1	1	1	1	1
13		2	2	2	2	2	2	2	2	1	1	1	1	1	1	1	1
14		1	1	1	1	2	2	2	2	1	1	1	1	1	1	1	1
15		1	1	1	1	2	2	2	2	1	1	1	1	1	1	1	1
16		1	1	1	1	2	2	2	2	2	2	2	2	1	1	1	1
17		1	1	1	1	2	2	2	2	1	1	1	1	1	1	1	1
18		**2**	**1**	**2**	**2**	2	2	2	2	1	1	1	1	1	1	1	1
19		1	1	1	1	2	2	2	2	1	1	1	1	**2**	**2**	**1**	**1**
20		**1**	**1**	**0**	**1**	2	2	2	2	1	1	1	1	1	1	1	1
21	1.25	1	1	1	1	2	2	2	2	1	1	1	1	1	1	1	1
22		1	1	1	1	2	2	2	2	1	1	1	1	1	1	1	1
23		1	1	1	1	2	2	2	2	1	1	1	1	1	1	1	1
24		1	1	1	1	2	2	2	2	1	1	1	1	1	1	1	1
25		1	1	1	1	2	2	2	2	1	1	1	1	1	1	1	1
26		1	1	1	1	2	2	2	2	1	1	1	1	1	1	1	1
27		1	1	1	1	2	2	2	2	1	1	1	1	1	1	1	1
28		1	1	1	1	2	2	2	2	1	1	1	1	1	1	1	1
29		1	1	1	1	2	2	2	2	1	1	1	1	1	1	1	1
30		**1**	**0**	**0**	**1**	2	2	2	2	1	1	1	1	1	1	1	1
31	0.625	**1**	**0**	**1**	**1**	1	1	1	1	2	2	2	2	**1**	**1**	**2**	**1**
32		1	1	1	1	2	2	2	2	2	2	2	2	1	1	1	1
33		1	1	1	1	2	2	2	2	1	1	1	1	1	1	1	1
34		1	1	1	1	2	2	2	2	1	1	1	1	1	1	1	1
35		1	1	1	1	2	2	2	2	1	1	1	1	1	1	1	1
36		1	1	1	1	2	2	2	2	2	2	2	2	1	1	1	1
37		1	1	1	1	1	1	1	1	**2**	**1**	**2**	**1**	**2**	**1**	**2**	**1**
38		1	1	1	1	2	2	2	2	**1**	**1**	**2**	**1**	1	1	1	1
39		1	1	1	1	2	2	2	2	2	2	2	2	1	1	1	1
40		1	1	1	1	2	2	2	2	1	1	1	1	1	1	1	1

Observer No.: 1 = researcher, 2 = surgical resident, 3 = board-certified surgeon, 4 = attending surgeon; (-) = no visualization available due to intraoperative circumstances. Cases of inter-observer variability in fluorescence signal evaluation are marked as bold on green background.

## Data Availability

This study was registered at ClinicalTrials.gov (NCT04709445). The study cluster “Perfusion Rate Assessment by Near-infrared Fluorescence in Gastrointestinal Anastomoses” is ongoing.
